# Supplementary material for: Molecular Characterization and Antibacterial Resistance Determination of Escherichia coli Isolated from Fresh Raw Mussels and Ready-to-Eat Stuffed Mussels: A Major Public Health Concern
Source: Pathogens. 2024 Jun 24;13(7):532. doi: 10.3390/pathogens13070532 (PMC11279604; doi:10.3390/pathogens13070532)
Supplement: Supplementary file 1 [file pathogens-13-00532-s001.zip › pathogens-3007966-supplementary.pdf]

Table S1. Regions, sampling points and types of samples were taken from four mussel processing and sale companies around the Marmara Sea.

Stuffed mussel production and sales companies and their harvesting locations	Sampling points and types of samples	Number of samples per month*
1st region (R1)-Balikesir (40°34'41.8"N 27°35'37.8" E)	Fresh raw mussel	25
	RTE stuffed mussel	25
2nd region (R2)-Mudanya (40°22'59.8"N 28°52'45.3" E)	Fresh raw mussel	25
	RTE stuffed mussel	25
3rd region (R3)-Gemlik (40°28'18.2"N 28°54'28.3"E)	Fresh raw mussel	25
	RTE stuffed mussel	25
4th region (R4)-Istanbul (40°59'31.1"N 29°00'46.1" E)	Fresh raw mussel (sampling point 1)	25
	Swab from knife (sampling point 2)	1
	Before the start of the step, swap sampling of the knife used in opening the shells	
	Shelling step (sampling point 3)	25
	Mussels whose shells were opened, internally cleaned and washed	
	Swab sample from food handlers' hand (sampling point 4)	1
	Before the start of the step, swap sampling was performed from the hands of the handler stuffing the pre-cooked rice	
	Stuffing with pre-cooked rice and spices (sampling point 5)	25
	Once the pre-cooked rice stuffing and shell closing stages were completed, raw stuffed mussels with their shells closed were sampled before cooking	
	Cooking with an aromatic blend of rice & spices (sampling point 6)	25
	Stuffed mussels after cooking	
	Portioning & packaging (sampling point 7)	25
	After portioning and packaging step, stuffed mussels were sampled before shipment	
	Shipment (sampling point 8)	25
	Stuffed mussels were sampled after shipment step (in the sale shop)	
	Selling (sampling point 9)	25
	RTE stuffed mussels were sampled at a point in time to sell	

RTE: Ready-to-eat; * Each 25 raw, semi-processed, or RTE stuffed mussel samples were taken on a single day and were considered as a single batch.

Table S2. *E. coli* isolates used in phenotypic antibacterial resistance and genetic diversity investigation.

Year	Month	Isolate No	Sampling region	Culture media	Samples
**2022**	**June**				
		7EM	R1	TBX	FRM
		30EM	R4	TBX	SP3
		31EM	R4	TBX	SP3
		46EM	R4	TBX	SP3
		47EM	R4	TBX	SP5
		76EM	R4	TBX	SP5
		164EM	R4	VRB	SP3
		194EM	R4	TBX	SP3
	**July**				
		8EM	R4	TBX	FRM
		86EM	R4	VRB	SP3
		87EM	R4	VRB	SP5
	**August**				
		10EM	R2	TBX	FRM
		11EM	R1	TBX	FRM
		21EM	R4	TBX	SP5
		192EM	R2	TBX	FRM
	**September**				
		12EM	R3	TBX	FRM
		13EM	R3	TBX	FRM
		25EM	R4	TBX	SP3
		26EM	R4	TBX	SP3
		28EM	R4	VRB	SP5
		39EM	R3	TBX	FRM
		40EM	R3	TBX	FRM
		44EM	R4	TBX	SP5
		45EM	R4	TBX	SP5
		108EM	R1	VRB	FRM
		138EM	R3	VRB	FRM
		145EM	R1	VRB	FRM
		156EM	R4	VRB	SP3
		166EM	R2	VRB	FRM
		182EM	R1	VRB	FRM
	**October**				
		16EM	R4	TBX	SP3
		17EM	R3	TBX	FRM
		18EM	R3	TBX	FRM
		20EM	R2	TBX	FRM
		22EM	R4	TBX	SP5
		23EM	R4	TBX	FRM
		24EM	R4	VRB	SP5
		41EM	R4	TBX	SP3
		42EM	R4	TBX	SP5
		43EM	R4	TBX	SP5
		123EM	R2	VRB	FRM
		140EM	R3	VRB	FRM
		144EM	R4	VRB	SP3
		178EM	R4	VRB	SP5
		188EM	R4	VRB	SP5
		198EM	R4	VRB	SP5
	**November**				
		32EM	R3	TBX	FRM
		33EM	R2	TBX	FRM

		34EM	R4	TBX	FRM
		48EM	R2	TBX	FRM
		49EM	R2	TBX	FRM
	**December**				
		35EM	R3	TBX	FRM
		50EM	R3	TBX	FRM
		51EM	R4	TBX	FRM
		52EM	R4	TBX	FRM
		53EM	R4	TBX	FRM
		54EM	R4	TBX	FRM
		55EM	R4	TBX	FRM
		60EM	R3	VRB	RTE-SM
		61EM	R3	VRB	FRM
		62EM	R3	VRB	FRM
		63EM	R3	VRB	FRM
**2023**	**January**				
		56EM	R2	TBX	FRM
		57EM	R2	TBX	FRM
		58EM	R2	TBX	FRM
		59EM	R2	TBX	FRM
		70EM	R2	TBX	FRM
		101EM	R4	TBX	FRM
	**February**				
		77EM	R4	TBX	SP3
		125EM	R4	TBX	FRM
		129EM	R4	TBX	SP3
		131EM	R4	TBX	SP3
		200EM	R1	VRB	FRM
	**March**				
		211EM	R1	TBX	FRM
	**April**	-	-	-	-
	**May**	-	-	-	-

FRM: Fresh raw mussel; RTE-SM, Ready-to-eat stuffed mussel; SP: Sampling point; -: *E. coli* was not detected in this month.

Table S3. Genes for phylogenetic groups detection of *E. coli* isolates.

	Isolate No	*arpA*	*TspE4.C2*	*yjaA*	*chuA*	Phylogroup *	Phylogroup **
**June-2022**	7EM	+	+	+	+	B2	Unknown
	30EM	+	+	+	+	B2	Unknown
	31EM	+	+	+	-	B1	Unknown
	46EM	+	-	+	-	A	A or C
	47EM	+	+	+	+	B2	Unknown
	76EM	+	-	-	-	A	A
	164EM	+	-	+	-	A	A or C
	194EM	+	-	-	-	B2	A
**July**	8EM	+	-	-	-	A	A
	86EM	+	-	-	-	A	A
	87EM	-	-	+	+	B2	B2
**August**	10EM	+	+	-	-	B1	B1
	11EM	+	+	+	-	B1	Unknown
	21EM	+	+	-	-	B1	B1
	192EM	-	-	-	-	A	Unknown
**September**	12EM	+	+	+	-	B1	Unknown
	13EM	+	-	-	-	A	A
	25EM	-	-	+	-	A	Clade I or II
	26EM	+	-	+	-	A	A or C
	28EM	+	+	+	-	B1	Unknown
	39EM	+	-	+	-	A	A or C
	40EM	+	+	+	-	B1	Unknown
	44EM	+	-	+	-	A	A or C
	45EM	+	-	-	-	A	A
	108EM	+	-	-	-	A	A
	138EM	+	-	-	-	A	A
	145EM	+	-	+	-	A	Clade I or II
	156EM	-	-	+	+	B2	B2
	166EM	-	-	+	+	B2	B2
	182EM	+	-	+	-	A	A or C
**October**	16EM	+	+	-	-	B1	B1
	17EM	+	+	-	-	B1	B1
	18EM	+	+	-	-	B1	B1
	20EM	-	-	-	+	D	F
	22EM	+	+	+	-	B1	Unknown
	23EM	+	+	+	-	B1	Unknown
	24EM	+	+	-	-	B1	B1
	41EM	+	+	+	+	B2	Unknown
	42EM	+	-	+	-	A	A or C
	43EM	+	+	-	-	B1	D or E
	123EM	+	-	-	-	A	A
	140EM	-	-	-	-	A	Unknown
	144EM	+	-	+	-	A	A or C
	178EM	+	-	+	-	A	A or C
	188EM	+	-	+	-	A	A or C
	198EM	+	-	+	-	A	A or C
**November**	32EM	+	+	-	+	D	Unknown
	33EM	+	-	-	-	A	A
	34EM	+	+	-	-	B1	B1
	48EM	-	+	+	+	B2	B2
	49EM	+	+	+	+	B2	Unknown
**December**	35EM	+	+	-	-	B1	B1
	50EM	+	+	-	+	D	Unknown

	51EM	+	+	-	-	B1	B1
	52EM	+	+	-	+	D	Unknown
	53EM	-	-	-	-	A	Unknown
	54EM	-	-	-	-	A	Unknown
	55EM	+	+	-	+	D	Unknown
	60EM	-	-	-	-	A	Unknown
	61EM	-	-	+	+	B2	B2
	62EM	+	-	+	+	B2	E or Clade I
	63EM	+	-	+	+	B2	E or Clade I
**January-2023**	56EM	+	+	-	-	B1	B1
	57EM	+	-	-	-	A	A
	58EM	+	-	-	-	A	A
	59EM	+	-	-	-	A	A
	70EM	+	-	-	-	A	A
	101EM	+	-	-	-	A	A
**February**	77EM	+	-	-	-	A	A
	125EM	-	-	-	-	A	Unknown
	129EM	-	-	-	-	A	Unknown
	131EM	-	-	-	-	A	Unknown
	200EM	-	-	-	-	A	Unknown
**March**	211EM	-	-	-	-	A	Unknown
**April** ***							
**May** ***							

-: This gene was not detected; * Characterized according to Clermont et al., 2000 **[22]**; ** Characterized according to Clermont et al., 2013 **[23]**; *** *E. coli* was not detected in this month.

Table S4. Antibacterial resistance and *ESBL* profile of *E. coli* isolates.

	Isolate No	*LVX*	*SXT*	*AMP*	*C*	*TE*	*CIP*	*STR*	*CTX*	*CDT* *	*DDST* *	*ESBL* *
**June-2022**	7EM	S	S	I	S	S	S	S	S			
	30EM	S	S	R	R	S	S	I	S			
	31EM	S	S	S	S	S	S	S	S			
	46EM	S	S	R	R	S	S	I	R	-	-	-
	47EM	S	S	S	S	S	S	S	S			
	76EM	S	S	R	S	S	S	S	S			
	164EM	S	S	S	S	S	S	S	R	-	-	-
	194EM	S	S	S	S	S	S	S	S			
**July**	8EM	S	R	R	S	S	I	R	R	-	-	-
	86EM	S	S	S	S	S	S	S	R	-	-	-
	87EM	S	S	S	S	S	S	I	S			
**August**	10EM	S	S	S	S	S	S	S	R	-	-	-
	11EM	S	S	R	S	R	S	S	S			
	21EM	S	S	R	S	S	S	S	S			
	192EM	S	S	S	S	S	S	S	S			
**September**	12EM	S	S	R	S	S	S	S	S			
	13EM	S	R	R	R	S	S	R	S			
	25EM	S	S	R	S	S	S	S	S			
	26EM	S	R	R	S	R	S	R	R	-	-	-
	28EM	S	S	S	S	S	S	S	S			
	39EM	S	S	S	S	S	S	R	S			
	40EM	S	S	S	S	S	S	S	S			
	44EM	S	R	R	S	S	I	R	S			
	45EM	S	S	I	S	S	S	S	S			
	108EM	I	R	S	S	S	R	S	S			
	138EM	S	S	R	S	S	S	R	S			
	145EM	S	R	R	S	R	S	S	R	-	-	-
	156EM	S	S	S	S	S	S	S	S			
	166EM	S	S	R	S	S	S	S	R	-	-	-
	182EM	S	S	S	S	R	S	S	S			
**October**	16EM	S	S	I	S	S	S	S	R	-	-	-
	17EM	S	R	I	S	S	S	S	S			
	18EM	S	S	S	S	S	S	S	S			
	20EM	S	S	S	S	S	S	S	S			
	22EM	S	S	S	S	S	S	S	S			
	23EM	S	R	R	R	R	S	I	S			
	24EM	S	S	R	S	S	S	S	S			
	41EM	S	S	R	S	S	S	S	R	+	+	+
	42EM	S	S	S	S	I	S	S	S			
	43EM	S	S	S	S	S	S	S	S			
	123EM	S	S	R	S	S	S	S	S			
	140EM	S	S	I	S	S	S	S	S			
	144EM	S	S	S	S	S	S	S	S			
	178EM	S	S	S	S	S	S	S	S			
	188EM	S	S	R	S	S	S	R	S			
	198EM	S	S	R	S	S	S	S	S			
**November**	32EM	S	S	S	S	S	S	S	S			
	33EM	S	S	R	S	R	S	R	R	-	-	-
	34EM	S	S	I	S	S	S	I	S			
	48EM	S	S	R	S	S	S	S	R	-	-	-
	49EM	S	S	S	S	S	S	S	S			
**December**	35EM	S	S	S	S	S	S	S	S			
	50EM	S	S	S	S	S	S	S	S			

	51EM	S	S	I	S	S	S	S	S			
	52EM	S	S	S	S	S	S	R	S			
	53EM	S	S	I	S	S	S	S	R	-	-	-
	54EM	S	S	S	S	S	S	R	R	-	-	-
	55EM	S	S	S	S	S	S	R	S			
	60EM	S	S	R	S	S	S	S	S			
	61EM	S	S	S	S	S	S	S	S			
	62EM	S	S	S	S	S	S	S	S			
	63EM	S	S	S	S	S	S	S	S			
	56EM	S	S	S	S	S	S	S	S			
	57EM	S	I	R	S	R	S	R	S			
**January-2023**	58EM	S	S	S	S	S	S	R	R	-	-	-
	59EM	R	R	R	R	I	R	R	S			
	70EM	R	R	R	R	I	R	R	S			
	101EM	S	S	S	S	S	S	S	S			
	77EM	S	S	S	S	S	S	S	S			
	125EM	S	S	S	S	S	S	S	S			
**February**	129EM	S	S	R	S	S	S	R	S			
	131EM	S	S	S	S	S	S	S	S			
	200EM	S	S	S	S	S	S	S	S			
**March**	211EM	S	S	S	S	S	S	S	S			
**April** **												
**May** **												

LVX: Levofloxacin (5 μg); SXT, trimethoprim-sulfamethoxazole (25 μg); AMP: ampicillin (10 μg), CL: chloramphenicol (30 μg); TE: tetracycline (30 μg); CIP: ciprofloxacin (5 μg); STR: streptomycin (10 μg); CTX: cephotaxime (30 μg); CDT: Combined disc test (according to the CLSI, 2023); DDST: Double-disc synergy test (according to the EUCAST, 2023); ESBL: Extended-spectrum beta-lactamase (according to the results of CDT and DDST tests); R: Resistant; I: Intermediate resistant; S: Sensitive; * In these columns - indicates that ESBL was negative; + indicates that ESBL was positive. If the cell is empty, it indicates that confirmation tests were not performed, as the CTX was negative; ** *E. coli* was not isolated in this month.

Table S5. Antibacterial resistance genes of *E. coli* isolates.

	Isolate No	*tetA*	*tetB*	*sul1*	*sul2*	*sul3*	*qnrA*	*qnrB*	*floR*	bla_{oxa}	bla_{TEM}	bla_{CTX}
**June-2022**	7EM	-	-	-	-	-	-	-	-	-	-	-
	30EM	-	+	-	-	-	-	-	-	+	-	-
	31EM	-	-	-	-	-	-	-	-	-	-	-
	46EM	-	+	-	-	-	-	-	-	+	-	-
	47EM	-	-	-	-	-	-	-	-	-	-	-
	76EM	+	-	-	-	-	-	-	-	-	-	-
	164EM	-	-	-	-	-	-	-	-	-	+	-
	194EM	-	-	-	-	-	-	-	-	-	-	-
**July**	8EM	+	-	+	+	-	-	+	-	-	+	-
	86EM	-	-	-	+	-	-	-	-	-	+	-
	87EM	-	-	-	-	-	-	-	-	-	-	-
**August**	10EM	-	-	-	-	-	-	-	-	-	-	-
	11EM	+	-	-	+	-	-	-	-	-	-	-
	21EM	-	-	-	-	-	-	-	-	-	-	-
	192EM	-	-	-	-	-	-	-	-	-	-	-
**September**	12EM	+	-	-	-	-	-	-	-	-	-	-
	13EM	-	-	-	-	-	-	-	-	-	-	-
	25EM	-	-	-	-	-	-	-	-	-	-	-
	26EM	-	-	-	+	-	-	-	-	-	+	-
	28EM	-	-	-	-	-	-	-	-	-	+	-
	39EM	+	-	-	-	+	-	-	-	-	-	-
	40EM	-	-	-	-	-	-	-	-	-	-	-
	44EM	+	-	-	+	-	-	-	-	-	-	-
	45EM	-	-	-	-	-	-	-	-	-	-	-
	108EM	+	-	-	-	-	-	-	-	-	-	-
	138EM	-	-	-	+	-	-	-	-	-	-	-
	145EM	-	+	-	-	-	-	-	-	-	-	-
	156EM	-	-	-	-	-	-	-	-	-	-	-
	166EM	-	-	-	-	-	-	-	-	-	+	-
	182EM	-	+	-	-	-	-	-	-	-	+	-
**October**	16EM	-	-	-	-	-	-	-	-	-	-	-
	17EM	-	-	-	-	-	-	-	-	-	-	-
	18EM	-	-	-	-	-	-	-	-	-	-	-
	20EM	-	-	-	-	-	-	-	-	-	-	-
	22EM	-	-	-	-	-	-	-	-	-	-	-
	23EM	-	+	+	-	-	-	-	-	+	-	-
	24EM	-	-	-	-	-	-	-	-	-	+	-
	41EM	-	-	-	-	-	-	-	-	-	-	-
	42EM	-	+	-	-	-	-	-	-	-	-	+
	43EM	-	-	-	-	-	-	-	-	-	-	-
	123EM	-	-	-	-	-	-	-	-	-	-	-
	140EM	-	-	-	-	-	-	-	-	-	-	-
	144EM	-	+	-	-	-	-	-	-	-	-	-
	178EM	-	+	-	-	-	-	-	-	+	-	-
	188EM	-	-	-	-	-	-	-	-	-	-	-
	198EM	-	-	-	-	-	-	-	-	-	-	-
**November**	32EM	-	-	-	-	-	-	-	-	-	+	-
	33EM	+	-	-	-	-	+	-	-	-	-	-
	34EM	-	-	-	-	-	-	-	-	-	-	-
	48EM	-	-	-	-	-	-	-	-	-	-	-
	49EM	-	-	-	-	-	-	-	-	-	-	-
**December**	35EM	-	-	-	-	-	-	-	-	-	-	-
	50EM	-	-	-	-	-	-	-	-	-	-	-

	51EM	-	-	-	-	-	-	-	-	-	-	-
	52EM	-	-	-	-	-	-	-	-	-	-	-
	53EM	-	-	-	-	-	-	-	-	-	-	-
	54EM	+	-	-	-	-	-	-	-	-	-	-
	55EM	-	-	-	-	-	-	-	-	-	-	-
	60EM	-	-	-	-	-	-	-	-	-	-	-
	61EM	-	-	-	-	-	-	-	-	-	-	-
	62EM	-	-	-	-	-	-	-	-	-	-	-
	63EM	-	-	-	-	-	-	-	-	+	-	-
**January-2023**	56EM	-	-	-	-	-	-	-	-	-	-	-
	57EM	-	+	-	+	-	-	-	-	-	+	-
	58EM	-	-	-	-	-	-	-	-	-	-	-
	59EM	-	+	+	+	+	-	-	-	-	-	-
	70EM	-	-	-	+	-	-	-	+	-	-	-
	101EM	-	-	-	-	-	-	-	-	-	-	-
**February**	77EM	-	-	-	-	-	-	-	-	-	-	-
	125EM	-	-	-	-	-	-	-	-	-	-	-
	129EM	-	-	-	-	-	-	-	-	-	-	-
	131EM	-	-	-	-	-	-	-	-	-	-	-
	200EM	-	-	-	-	-	-	-	-	-	-	-
**March**	211EM	-	-	-	-	-	-	-	-	-	-	-
**April** *												
**May** *												

-: This gene was not detected; +: This gene was detected; * *E. coli* was not isolated in this month.
